# Wave turbulence and cortical dynamics

**DOI:** 10.3389/fncom.2026.1682176

**Published:** 2026-03-20

**Authors:** Gerald K. Cooray

**Affiliations:** 1 Clinical Neuroscience, Karolinska Institutet, Stockholm, Sweden; 2 GOS-ICH, University College of London, London, United Kingdom

**Keywords:** cortical tissue, neural fields, self organized criticality, spectral dynamics, turbulent flow

## Abstract

Cortical activity recorded through EEG and MEG reflects complex dynamics that span multiple temporal and spatial scales. Spectral analyses of these signals consistently reveal power-law behavior, a hallmark of turbulent systems. In this paper, we derive a kinetic equation for neural field activity based on wave turbulence theory, highlighting how quantities such as energy and pseudo-particle density flow through wave-space (*k*-space) via direct and inverse cascades. We explore how different forms of nonlinearity–particularly 3-wave and 4-wave interactions–shape spectral features, including harmonic generation, spectral dispersion, and transient dynamics. While the observed power-law decays in empirical data are broadly consistent with turbulent cascades, variations across studies—such as the presence of dual decay rates or harmonic structures—point to a diversity of underlying mechanisms. We argue that although no single model fully explains all spectral observations, key constraints emerge: namely, that cortical dynamics exhibit features consistent with turbulent wave systems involving both single and dual cascades and a mixture of 3- and 4-wave interactions. This turbulence-based framework offers a principled and unifying approach to interpreting large-scale brain activity, including state transitions and seizure dynamics.

## 1 Introduction

Cortical activity at small spatial and temporal scales often exhibits oscillatory transients with a complex and variable mixture of frequencies (Buzsáki and Vöröslakos, 2023; Buzsáki, 2006). This activity has been effectively modeled using nodal approaches, where each node follows neural mass dynamics with a complex interaction between nodes. Neural mass models are widely used to simulate brain signals such as EEG and MEG by modeling interactions between excitatory and inhibitory neural populations (David and Friston, 2003; Byrne et al., 2020; Jansen and Rit, 1995). At a more mesoscopic level, the cortex, with its convoluted sheet-like geometry, is well represented as a field defined over a two-dimensional surface. Many models of cortical dynamics describe propagating waves of activity, often interacting non-linearly across both space and time–or equivalently, across time and frequency domains (Cook et al., 2022; Coombes, 2005; Lopes da Silva et al., 1974; Bressloff, 2011; Wilson and Cowan, 1973).

Several empirical features of cortical activity constrain the development of such models, including signal decay and finite wave transmission speeds. Despite these constraints, the dynamics remain highly complex, as evidenced in electroencephalogram (EEG) recordings, particularly in subjects with neurological disorders such as epilepsy (Jirsa et al., 2014; Lagarde et al., 2019).

A prominent and consistent finding in recordings of cortical activity—whether at the level of local field potentials (LFPs) from neural populations, intracranial electrodes sampling from regions of approximately 1*mm*^3^, or scalp electrodes measuring from several *cm*^2^—is the presence of a spectral power-law decay in signal power across frequencies (He, 2014). This $\frac{1}{f^{a}}$ scaling is a hallmark of turbulent flow dynamics (Donoghue et al., 2022).

Turbulence has been extensively studied in fluid dynamics, with a rich literature describing both experimental observations and rigorous theoretical frameworks derived from fundamental equations such as the Navier-Stokes equation. This non-linear partial differential equation exhibits turbulent solutions under certain conditions, where perturbed equilibria evolve into steady states characterized by a constant flux of energy or other conserved quantities. The mathematical description of such turbulent states was pioneered by Richardson and Kolmogorov (Kolmogorov, 1991; Frisch and Kolmogorov, 1995), whose work accurately predicted the spectral features of turbulent energy cascades.

It is striking that neural field dynamics—despite their differing physical foundations—also exhibit spectral features reminiscent of those seen in turbulence. This resemblance can be more naturally understood in the framework of wave turbulence (or weak wave turbulence), a well-developed theory describing non-linear interactions between waves that generate turbulent-like spectral distributions (Nazarenko, 2011; Zakharov et al., 1992).

Recent work has proposed that cortical dynamics may follow principles of turbulent wave activity, offering a potential explanation for the observed power-law behavior in cortical spectra (Deco and Kringelbach, 2020; Deco et al., 2025). Such turbulence may shape numerous aspects of brain function, including rapid transitions between distinct oscillatory states. Notably, epileptic seizures–sudden and dramatic disruptions of cortical activity–are accompanied by characteristic changes in time-frequency dynamics (Jirsa et al., 2014; Lagarde et al., 2019). Scale-free dynamics and long-range correlations, both hallmarks of turbulent systems, are also central features of dynamic criticality (Frisch and Kolmogorov, 1995). This study engages directly with the mechanisms through which criticality may arise in neural systems (O'Byrne and Jerbi, 2022), while also offering mechanistic insights into how such criticality might influence observed patterns of neural activity.

In this paper, we begin with a model of neural activity and derive from it the characteristic behavior of turbulent flow. We then compare these theoretical predictions with experimental results from cortical recordings. We show that many empirical findings can be plausibly explained by assuming that cortical activity is governed by wave turbulence.

## 2 Model

We begin with the cortical dynamics previously analyzed in several studies (Cooray et al., 2023, 2024, 2025). The complete model examined in this paper is, overall, non-conservative, meaning that it includes energy dissipation. Nonetheless, it is built around a conservative core that can produce oscillations, as outlined in Sections 2-4. In Sections 4-5, the non-conservative features of the model are incorporated so that turbulence-based predictions can be derived. Although the model has a hierarchical structure, energy loss occurs at every level of the dynamics and is primarily introduced as a computational or analytical device.

### 2.1 The equation of motion

The evolution of cortical activity is governed by the following equation (Equation 1):


$$\begin{array}{l} \begin{array}{c} i\partial_{t}\phi \left(\text{r},t\right)=F\left(\phi \left({\text{r}}^{\prime },t^{\prime }\right)\right) \end{array} \end{array}$$ (1)

The neural field variable, ϕ, is a complex scalar where the real and imaginary components reflect activity in excitatory and inhibitory layers of the cortex. The field ϕ is defined on the cortical surface, which is a two-dimensional manifold (i.e., a curved surface). The functional *F*, maps the neural field activity across all spatial and earlier temporal points to a complex scalar that describes the change at a given point of time and location, which represents the most general form used to characterize cortical field dynamics. The functional can be expanded as a set of integrals with spatial and temporal kernels (first, second and third order terms are included). Note that several other kernel integrals could be included but the terms included are those which stabilize oscillations or equivalently integrable components, Equation 2.


$$\begin{array}{l} i\partial_{t}\phi \left(\text{r},t\right)=\int K_{0}\left(\text{r},{\text{r}}_{1}\right)\phi \left({\text{r}}_{1},t_{1}\right)dt_{1}dr_{1}+\\ +\int K_{1}\left(\text{r},{\text{r}}_{1},{\text{r}}_{2},\right)\phi \left({\text{r}}_{1},t_{1}\right)\phi \left({\text{r}}_{2},t_{2}\right)dt_{1}dt_{2}dr_{1}dr_{2}+...\\ +\int K_{1}^{*}\left(\text{r},{\text{r}}_{1},{\text{r}}_{2}\right)\phi \left({\text{r}}_{1},t_{1}\right)\phi^{*}\left({\text{r}}_{2},t_{2}\right)dt_{1}dt_{2}dr_{1}dr_{2}+...\\ +\int K_{2}\left(\text{r},{\text{r}}_{1},{\text{r}}_{2},{\text{r}}_{3}\right)\phi \left({\text{r}}_{1},t_{1}\right)\phi \left({\text{r}}_{2},t_{2}\right)\phi^{*}\left({\text{r}}_{3},t_{3}\right)dt_{1}dt_{2}dt_{3}dr_{1}dr_{2}dr_{3}\\ +... \end{array}$$ (2)

The kernels characterize the intrinsic (or local) memory of the system (although a global memory, set by the system's dynamics and not explicit in the equation of motion, may also exist). The local memory is constrained by the half-life of neuronal activation triggered by a single action potential (Wilson and Cowan, 1972). This duration depends on the synaptic transmitter type and is taken here to be 5-10 ms (Destexhe et al., 1998). Over this interval, the time integral can be approximated by the time-averaged value of the neural field using the “tilde” notation, $\tilde{\phi }$. In practice, this implies an upper frequency limit of about 100–200 Hz, beyond which this approximation—and thus the inferred dynamics—breaks down, Equation 3.


$$\begin{array}{l} i\partial_{t}\phi \left(\text{r},t\right)=\int K_{0}\left(\text{r},{\text{r}}_{1}\right)\tilde{\phi }\left({\text{r}}_{1},t\right)dr_{1}+\\ \;+\int K_{1}\left(\text{r},{\text{r}}_{1},{\text{r}}_{2}\right)\tilde{\phi }\left({\text{r}}_{1},t\right)\tilde{\phi }\left({\text{r}}_{2},t\right)dr_{1}dr_{2}+...\\ \;+\int K_{1}^{*}\left(\text{r},{\text{r}}_{1},{\text{r}}_{2}\right)\tilde{\phi }\left({\text{r}}_{1},t_{1}\right){\tilde{\phi }}^{*}\left({\text{r}}_{2},t\right)dr_{1}dr_{2}+...\\ \;+\int K_{2}\left(\text{r},{\text{r}}_{1},{\text{r}}_{2},{\text{r}}_{3}\right)\tilde{\phi }\left({\text{r}}_{1},t\right)\tilde{\phi }\left({\text{r}}_{2},t\right){\tilde{\phi }}^{*}\left({\text{r}}_{3},t\right)dr_{1}dr_{2}dr_{3}+... \end{array}$$ (3)

In the remainder of this paper, we omit the use of the “tilde” notation, assuming that time averaging has already been carried out. As a starting point for our analysis, we represent the dynamics in terms of the Fourier transform of the field ϕ together with the kernel functions, Equation 4.


$$\begin{array}{l} \;\int \phi \left(\text{r},t\right)e^{-i\text{k}.\text{r}}=\psi \left(\text{k},t\right)\\ \;\int K_{0}\left(\text{r}\right)e^{-i\text{k}.\text{r}}=\omega \left(\text{k}\right)\\ \;\int K_{1}\left({\text{r}}_{1},{\text{r}}_{2}\right)e^{-i{\text{k}}_{1}.{\text{r}}_{1}-i{\text{k}}_{2}.{\text{r}}_{2}}=K_{1}\left({\text{k}}_{1},{\text{k}}_{2}\right)\\ \int K_{2}\left({\text{r}}_{1},{\text{r}}_{2},{\text{r}}_{3}\right)e^{-i{\text{k}}_{1}.{\text{r}}_{1}-i{\text{k}}_{2}.{\text{r}}_{2}-i{\text{k}}_{3}.{\text{r}}_{3}}=K_{2}\left({\text{k}}_{1},{\text{k}}_{2},{\text{k}}_{3}\right) \end{array}$$ (4)

The equation motion will then be given by the following:


$$\begin{array}{l} i\partial_{t}\psi \left(\text{k},t\right)=\int K_{0}\left(\text{k},{\text{k}}_{1}\right)\psi \left({\text{k}}_{1},t\right)\delta \left(\text{k}-{\text{k}}_{1}\right)dk_{1}+...\\ +\int K_{1}\left(\text{k},{\text{k}}_{1},{\text{k}}_{2}\right)\psi \left({\text{k}}_{1},t\right)\psi \left({\text{k}}_{2},t\right)\delta \left(\text{k}-{\text{k}}_{1}-{\text{k}}_{2}\right)dk_{1}dk_{2}+...\\ +\int K_{1}^{*}\left(\text{k},{\text{k}}_{1},{\text{k}}_{2}\right)\psi \left({\text{k}}_{1},t\right)\psi^{*}\left({\text{k}}_{2},t\right)\delta \left(\text{k}+{\text{k}}_{2}-{\text{k}}_{1}\right)dk_{1}dk_{2}+...\\ +\int K_{2}\left(\text{k},{\text{k}}_{1},{\text{k}}_{2},{\text{k}}_{3}\right)\psi^{*}\left({\text{k}}_{1},t\right)\psi \left({\text{k}}_{2},t\right)\psi \left({\text{k}}_{3},t\right)\delta (\text{k}+{\text{k}}_{1}-{\text{k}}_{2}\\ -{\text{k}}_{3})dk_{1}dk_{2}dk_{3}+... \end{array}$$ (5)

### 2.2 Hamiltonian formalism

As noted above, the equation of motion was divided into components that were integrable and those that were not. The separation between these two was relatively straightforward: the condition for integrability is that the Hamiltonian (defined below) remains a real-valued function, which, for the class of equations considered here, corresponds to real-valued combinations of the neural field ψ and its complex conjugate $\bar{\psi }$. Any term that yields a complex-valued contribution leads to non-integrable behavior, such as energy dissipation. The analysis was done for the integrable terms of the dynamics, however, these non-integrable components are important and will become particularly relevant when steady-state solutions are analyzed. The system is approximated as an integrable one, comprising a linear wave equation supplemented by non-linear interactions between frequencies. The turbulence will be governed by this inter-frequency coupling, giving rise to a complex dynamical system. In addition, steady-state turbulent solutions will be estimated, which necessitates that at least some components of the dynamics are non-integrable. Such non-integrability is required to introduce energy sinks and sources within the dynamics, which are crucial for the formation of a steady-state solution. A Hamiltonian framework can be formulated to analyse the integrable portion of the dynamics. Noting that ψ is complex valued, the dynamics can be written as follows, Equation 6:


$$\begin{array}{l} i\partial_{t}\psi \left(\text{k},t\right)=\frac{\delta H\left(\psi \right)}{\delta \psi^{*}} \end{array}$$ (6)

We begin by expanding the functional H, which will be a real function of the neural field and its conjugate, Equation 7.


$$\begin{array}{l} H=H_{0}+H_{int} \end{array}$$ (7)

This set of equations allows us to analyse the dynamics using perturbation theory around the solution corresponding to $H_{0}$. A Hamiltonian such as $H_{0}$, which leads to wave equations, can often be written, after a suitable canonical transformation of the ψ variables, in a simplified form amenable to analysis, Equations 8 and 9.


$$\begin{array}{l} H_{0}=\int d\text{k}\omega \left(\text{k}\right)\psi \psi^{*} \end{array}$$ (8)

We then get the linearised solutions.


$$\begin{array}{l} i\partial_{t}\psi \left(\text{k},t\right)=\omega \left(\text{k}\right)\psi \left(\text{k},t\right)\\ \;\psi =Ae^{-i\omega \left(\text{k}\right)t}\\ \;\phi =A^{\prime }e^{-i\left(\omega \left(\text{k}\right)t\pm \text{k}.\text{r}\right)} \end{array}$$ (9)

If the ω-term is independent of **k** we get the Klein-Gordon equation which was derived in Cooray et al. (2024). In general we will have the following equation (Equation 10):


$$\begin{array}{l} \omega \left(\text{k}\right)=C|\text{k}{|}^{\alpha } \end{array}$$ (10)

There is empirical evidence suggesting that the value of α is greater than 1, indicating decaying waves. This leads to dispersion of wave packets on the cortical surface, as higher-frequency components travel faster than lower-frequency ones, resulting in attenuation and spreading of the wave packet over time. To simplify the notation, we will omit writing *t* explicitly in what follows.

### 2.3 Non-linear dynamics

The interaction term $H_{\text{int}}$ introduces non-linear coupling between waves. It is possible to derive kinetic equations that describe the flow of waves in *k*-space. By expanding the interaction functional in terms of contributions involving an increasing number of interacting waves, we obtain a series of interaction terms. In this work, we focus on three-wave and four-wave interactions. It has been shown that the dominant dynamics are typically captured by the lowest-order non-vanishing interaction term. For a detailed derivation of the kinetic equations, we refer the reader to the following standard texts (Zakharov et al., 1992; Nazarenko, 2011). The interaction Hamiltonian is specified in Equation 11.


$$\begin{array}{l} H_{int}=\int dk_{1}dk_{2}dk_{3}[K_{1}(k_{1},k_{2},k_{3})(\psi^{*}(k_{1})\psi (k_{2})\psi (k_{3})\delta (k_{1}\\ -k_{2}-k_{3}))]+...\\ \int dk_{1}dk_{2}dk_{3}[K_{1}^{*}(k_{1},k_{2},k_{3})(\psi^{*}(k_{1})\psi^{*}(k_{2})\psi (k_{3})\delta (k_{1}+k_{2}\\ -k_{3}))]+...+\int dk_{1}dk_{2}dk_{3}k_{4}\\ {}[K_{2}(k_{1},k_{2},k_{3},k_{4})(\psi^{*}(k_{1})\psi^{*}(k_{2})\psi (k_{3})\psi (k_{4})\delta (k_{1}+k_{2}\\ -k_{3}-k_{4}))] \end{array}$$ (11)

**Figure 1:**
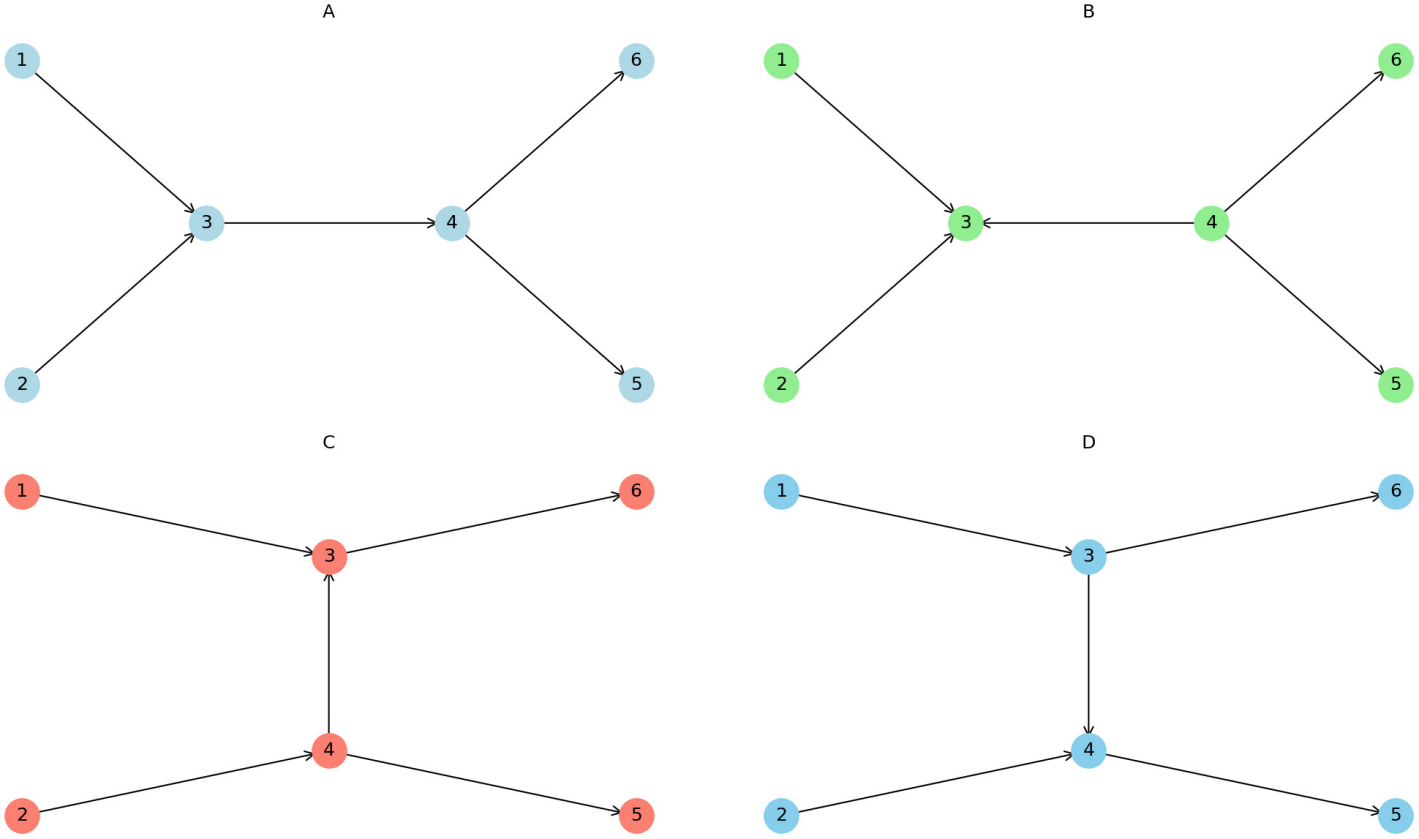
In the case of a Hamiltonian with three-wave interactions, the first-order contribution vanishes due to phase averaging of the interacting waves. However, the second-order terms yield a non-zero contribution, effectively resulting in a four-wave interaction mediated by a virtual (or forced) intermediate wave. **(A)** Two directly interacting waves (1, 2) creating two output waves (5, 6). **(B)** Similar to **(A)** but with the virtual intermediate wave moving in the opposite direction. **(C)** Two waves interacting (1, 2) with a virtual intermediate wave giving two resulting waves (5, 6). **(D)** Similar to **(C)** but with the intermediate wave moving in the opposite direction.

The different terms of the Hamiltonian function describe different types of wave interactions. The first two integrals describe 3 wave interactions, $H_{3}$. These waves are valid only for waves with a decay in energy, i.e. if α > 1. This process will be a 2 → 1 or a 1 → 2 wave process. Later on we will need to investigate a statistical collection of neural fields where averaging over the phase variable is required. The above processes of unequal number of waves before and after the interaction will lead to non-zero contributions if paired with the appropriate conjugate term. This would be due to higher order perturbation terms from the $H_{3}$ interaction (Figure 1). We have for 3-wave interactions the following equation of motion, Equation 12.


$$\begin{array}{l} i\frac{\partial \psi \left(\text{k}\right)}{\partial t}=\frac{\delta \left(H_{0}+H_{3}\right)}{\delta \psi^{*}}=\omega_{\text{k}}\psi \left(\text{k}\right)+...\\ \;\int [\frac{1}{2}K_{1}\left(\text{k},{\text{k}}_{1},{\text{k}}_{2}\right)\psi \left({\text{k}}_{1}\right)\psi \left({\text{k}}_{2}\right)\delta \left(\text{k}-{\text{k}}_{1}-{\text{k}}_{2}\right)+...\\ \;+K_{1}^{*}\left({\text{k}}_{1},\text{k},{\text{k}}_{2}\right)\psi \left({\text{k}}_{1}\right)\psi^{*}\left({\text{k}}_{2}\right)\delta \left({\text{k}}_{1}-\text{k}-{\text{k}}_{2}\right)]d{\text{k}}_{1}d{\text{k}}_{2} \end{array}$$ (12)

The four-wave Hamiltonian, $H_{4}$, is defined by the final integral in Equation 11 and leads to the set of equations of motion presented in Equation 13.


$$\begin{array}{l} i\frac{\partial \psi \left(\text{k}\right)}{\partial t}=\frac{\delta \left(H_{0}+H_{4}\right)}{\delta \psi^{*}}=\omega_{\text{k}}\psi \left(\text{k}\right)+...\\ \;+\int [K_{2}\left(\text{k},{\text{k}}_{1},{\text{k}}_{2},{\text{k}}_{3}\right)\psi^{*}\left({\text{k}}_{1}\right)\psi \left({\text{k}}_{2}\right)\psi \left({\text{k}}_{3}\right)\\ \;\delta \left(\text{k}+{\text{k}}_{1}-{\text{k}}_{2}-{\text{k}}_{3}\right)]d{\text{k}}_{1}d{\text{k}}_{2}d{\text{k}}_{3} \end{array}$$ (13)

Another issue concerning the kernels in the equations of motion that has not yet been addressed is the spatial range of the connections. Empirical studies, particularly in the human brain, demonstrate the coexistence of long-range connections with dense local connectivity. Substantial evidence indicates that neuronal activity relies on a combination of local and long-range connections. Long-range connectivity may be incorporated either as an external forcing term in the cortical dynamics or modeled explicitly. Several studies have incorporated long-range connectivity via periodic or quasi-periodic boundary conditions (Bressloff and Cowan, 2002; Bressloff, 2003; Laing and Troy, 2003; Cooray et al., 2024). These analyses demonstrate that the field dynamics become more elaborate when such distant connections are included, with repeating patterns emerging from complex interactions. However, the majority of these interactions link dynamical states that are separated by comparatively large cortical distances, implying that local activity is only weakly influenced. In this work, long-range connections are instead modeled as external, independent forcing terms. This assumption of independent forcing restricts the cortical spatial extent that can be faithfully described within the turbulence framework. A plausible maximal distance depends on the specific cortical area under consideration, but estimates typically lie in the range of 10–50 mm (Oligschläger et al., 2017; Sepulcre et al., 2010; Hagmann et al., 2008). This raises the subsequent question of whether the cortical patch being modeled is sufficiently large for turbulent properties to manifest, i.e. if there are enough scales for the turbulent processes to occur. There will be an upper frequency cutoff determined by the physical size of the neuronal units that generate the neural activity. For example, a unit size of 0.1 mm combined with a wave propagation speed of 10 mm/s yields an upper cutoff of about 100 Hz. Conversely, the lower frequency cutoff is set by the largest cortical region in which local connectivity predominates, taken here as about 10 mm, corresponding to roughly 1 Hz. Together, these bounds define a set of characteristic scales within which the dynamics can operate.

## 3 Statistical approximation

In Section 2, we derived the dynamic models governing the wave-like components that describe neural field activity. These waves are part of large-scale systems involving a multitude of distinct wave modes, where a statistical description is often necessary to capture the overall dynamics. In such systems, both the amplitude and phase of the **k**-waves may vary. We will average over the phase to get a statistical variable of the neural field and neural field correlation functions, as defined in (Equations 14, 15). This approximation is significant because it alters the dynamics observed with macroelectrodes: the oscillations of individual waveforms are no longer evident. Instead, resonances arise–multi-wave interactions that come to dominate the dynamics. In the next section, using a statistical approximation, it is shown that multi-wave interactions produce resonances that control the system's behavior, even though the interaction Hamiltonian is added only as a perturbation to the original dynamics. This resulting activity is nonlinear, despite the fact that the underlying model from which it is derived (prior to applying the statistical approximation) is governed primarily by linear dynamics. The full framework is in fact a statistical model, built on the assumption that the activity recorded over the cortex arises from a large ensemble of neuronal units. This assumption has a long history in the analysis of neural field activity and effectively averages out the randomness in both the connectivity network and the intrinsic dynamics of single-neuron firing (Ventriglia, 1973, 1983; Wilson and Cowan, 1972; Amari, 1977; Sbitnev, 1975; Fischer, 1973).


$$\begin{array}{l} \int \psi \left(\text{k}\right)d\Omega =\langle \psi \left(\text{k}\right)\rangle =0 \end{array}$$ (14)

The correlations between wavefunctions to lowest order are shown in Equation 15. When averaging over phases at a specific location on the cortical sheet, only the conjugate terms survive, leading to resonances. This is what drives the dynamics—initially described as a linear perturbation in Equation 7—into a regime where three- or four-wave interactions become the dominant contributors.


$$\begin{array}{l} \;\langle \psi \left(\text{k}\right)\psi \left({\text{k}}^{\prime }\right)\rangle =0\\ \;\langle \psi \left(\text{k}\right)\psi^{*}\left({\text{k}}^{\prime }\right)\rangle =n\left(\text{k}\right)\delta \left(\text{k}-{\text{k}}^{\prime }\right)\\ \langle \psi^{*}\left({\text{k}}_{1}\right)\psi^{*}\left({\text{k}}_{2}\right)\psi \left({\text{k}}_{3}\right)\psi \left({\text{k}}_{4}\right)\rangle =\\ \;n\left({\text{k}}_{1}\right)n\left({\text{k}}_{2}\right)[\delta \left({\text{k}}_{1}-{\text{k}}_{3}\right)\delta \left({\text{k}}_{2}-{\text{k}}_{4}\right)+\delta \left({\text{k}}_{1}-{\text{k}}_{4}\right)\delta \left({\text{k}}_{2}-{\text{k}}_{3}\right)] \end{array}$$ (15)

The evolution of **n**, the wave amplitude, is obtained through a detailed derivation of the equations of motion (Equations 12, 13). This full derivation is presented in Zakharov et al. (1992). The final outcome of that analysis is summarized in Equations 16, 17.


$$\begin{array}{l} \frac{\partial n\left(\text{k}\right)}{\partial t}=\\ \pi \int [|K_{1}\left(\text{k},{\text{k}}_{1},{\text{k}}_{1}\right){|}^{2}N_{1}\left(\text{k},{\text{k}}_{1},{\text{k}}_{2}\right)\\ +2|K_{1}\left({\text{k}}_{1},\text{k},{\text{k}}_{1}\right){|}^{2}N_{1}\left({\text{k}}_{1},\text{k},{\text{k}}_{2}\right)]d{\text{k}}_{1}d{\text{k}}_{2} \end{array}$$ (16)

$$\begin{array}{l} \frac{\partial n_{\text{k}}}{\partial t}=\frac{\pi }{2}\int [|K_{2}\left(\text{k},{\text{k}}_{1},{\text{k}}_{2},{\text{k}}_{3}\right){|}^{2}N_{2}\left(\text{k},{\text{k}}_{1},{\text{k}}_{2},{\text{k}}_{3}\right)]d{\text{k}}_{1}d{\text{k}}_{2}d{\text{k}}_{3} \end{array}$$ (17)

Where $N_{1}$ and $N_{2}$ are defined in Equations 18, 19.


$$\begin{array}{l} N_{1}(k,k_{1},k_{2})=\\ \left[n(k_{1})n(k_{2})-n(k)(n(k_{2})+n(k_{1})))\delta (k-k_{1}-k_{2}\right]\\ \delta (\omega_{k}-\omega_{1}-\omega_{2}) \end{array}$$ (18)

$$\begin{array}{l} N_{2}(k,k_{1},k_{2},k_{3})=\\ {}[n(k_{2})n(k_{3})(n(k_{1})+n(k))-n(k_{1})n(k)(n(k_{2})\\ +n(k_{3})))\delta (k+k_{1}-k_{2}-k_{3}]\delta (\omega_{k}+\omega_{1}-\omega_{2}-\omega_{3}) \end{array}$$ (19)

The integrals (Equations 16, 17) will be denoted by *I*(*k, t*), with the type of Hamiltonian (three-wave or four-wave) implicitly understood from the context. The integral *I* characterizes how the dynamics are distributed over different frequencies. In steady state, the dynamics produce an integral *I* that does not depend on the time variable *t*.

## 4 Wave turbulence

We now describe various theoretical possibilities for steady-state spectral features.

### 4.1 Conserved quantities and currents

Equations 16, 17 characterize the effects of three-wave and four-wave interactions on the wave action, or equivalently, the flux of wave energy through *k*-space. The steady state is defined by the condition $\frac{\partial n}{\partial t}=0$, indicating a constant spectral composition. Furthermore, in an equilibrium state, the wave action remains unchanged by definition. This condition allows for the derivation of several expressions that constrain the dynamics of the system, often referred to as constants of motion, Equations 20, 21, 22. We denote the total energy of the system by *E*, and the total wave action by *N*.


$$\begin{array}{l} E=\int \omega n\left(\text{k},t\right)d\text{k}\\ N=\int n\left(\text{k},t\right)d\text{k} \end{array}$$ (20)

Each of these conserved quantities is associated with a corresponding conserved current, expressed by the following formula, where *p* denotes a source term for *n*.


$$\begin{array}{l} \frac{\partial n}{\partial t}+\nabla \cdot p=0 \end{array}$$ (21)

The negative of the divergence of the current is given by the collision integral derived in Section 3.


$$\begin{array}{l} \frac{\partial n}{\partial t}=I \end{array}$$ (22)

### 4.2 Biophysical context of the conserved quantities and currents

To obtain strictly conserved quantities for neural fields, one must assume that the underlying dynamics are integrable. This is a fairly strong requirement, and empirical data suggest that it does not hold for neural fields. Nonetheless, as outlined in Section 2, the dynamics can be decomposed into conservative and non-conservative parts. This viewpoint allows us to distinguish strongly “non-conservative” models—comprising only non-conservative terms—from weakly “non-conservative” models, which include just a small number of non-conservative contributions in addition to a larger set of conservative ones. Empirically, one can estimate the half-life of activity in different frequency bands to quantify how rapidly the system's power decays. This can be approximated using evoked responses following brain stimulation. In humans, reproducible signals are observed for up to 1 s across both auditory and electrically evoked responses (Al-Sadek et al., 2025; Nourski, 2017). Over these timescales, the aforementioned quasi-conserved quantities remain approximately unchanged: namely, the total rms of the spectral power (corresponding to the wave-action component) and the mean frequency of the spectrum (corresponding to the energy component).

### 4.3 Equilibrium solutions

Equilibrium solutions will mainly serve as a contrast to the central phenomenon under investigation, the non-equilibrium solutions. The assumptions underlying equilibrium solutions stand in sharp contrast to what is observed in empirical data. In particular, the solution presupposes purely conservative dynamics at all frequencies, a claim that lacks empirical support. Moreover, constructing an equilibrium solution would require that the cortex be driven via long-range connections that are exquisitely tuned so that the energy loss in each frequency component of the spectrum is exactly compensated, given the absence of exchange between different frequency bands. While this is a theoretical possibility, it is extremely improbable in practice. The solution itself, however, follows straightforwardly from imposing conservation of entropy (disorder) and energy, as specified in Equation 23.


$$\begin{array}{l} S=\int \ln\;n\left(\text{k},t\right)d\text{k} \end{array}$$ (23)

Varying the entropy while keeping the energy constant yields the equilibrium solutions, where μ is a Lagrangian constant (Equation 24)


$$\begin{array}{l} \delta \left(S+\mu E\right)=\delta [\int \ln\;n\left(\text{k},t\right)d\text{k}+\mu \int \omega n\left(\text{k},t\right)d\text{k}] \end{array}$$ (24)

The equilibrium solution of the system is given in Equation 25.


$$\begin{array}{l} \;0=\frac{1}{n\left(\text{k},t\right)}+\mu \omega \\ n\left(\text{k},t\right)=-\frac{1}{\mu \omega } \end{array}$$ (25)

### 4.4 Non-equilibrium solutions—Kolmogorov solutions

Non-equilibrium steady states, also referred to as Kolmogorov solutions, can be obtained from the equations of motion (Equations 16, 17). The derivations presented in this section yield predictions that can be directly compared with experimentally observed cortical activity; see Section 4.7. These steady states will be shown to display a power-law decay as a function of frequency. The corresponding decay rate, or decay exponent, can be estimated under suitable approximations. In particular, when the system is both isotropic and scale invariant, these decay exponents can be determined. We adopt the definition of scale invariance given in Equation 26.


$$\begin{array}{l} \left|K\left(\lambda k_{1},\lambda k_{2},\lambda k_{3}\right)|=\lambda^{m}|K\left(k_{1},k_{2},k_{3}\right)\right| \end{array}$$ (26)

The energy flux will be given by Equation 27, where the flow of waves is multiplied by the temporal frequency, ω to get the energy flux


$$\begin{array}{l} \frac{dP\left(k\right)}{dk}=-{\left(2k\right)}^{d-1}\pi \omega \left(k\right)I\left(k\right) \end{array}$$ (27)

This relation can be integrated over the k-variable.


$$\begin{array}{ll} P\left(k\right) & ≃\int_{0}^{k}dk{\left(2k\right)}^{d-1}\pi \omega \left(k\right)\frac{\partial n}{\partial t}=\int_{0}^{k}dk{\left(2k\right)}^{d-1}\pi \omega \left(k\right)I\left(k\right) \end{array}$$ (28)

Equation 28 can be used to estimate a power law distribution of *n* over *k* defined in Equation 29.


$$\begin{array}{l} n\left(k\right)=Ak^{-s} \end{array}$$ (29)

By applying dimensional analysis, one can estimate steady-state solutions (Kolmogorov states), in which the integral *I* (Equation 16, 17) carries dimensions in *k* as specified in Equation 30.


$$\begin{array}{l} I\approx \int |V{|}^{2}n^{2}\delta \left(k\right)\delta \left(\omega \right)dk^{2}\\ \;\approx k^{2m-2s-d-\alpha +2d} \end{array}$$ (30)

We get the following dimensions for the energy flux, *P*(*k*), Equation 31.


$$\begin{array}{l} P\left(k\right)\approx \int_{0}^{k}dk{\left(2k\right)}^{d-1}\pi \omega \left(k\right)\frac{\partial n}{\partial t}\\ \;\approx k^{d+\alpha }k^{2m-2s+d-\alpha }\\ \;=k^{2\left(m+d-s\right)} \end{array}$$ (31)

The wave action flux, *Q*(*k*), will give a similar equation, i.e., the flow of the waves at a given **k**, Equations 32, 33.


$$\begin{array}{l} Q\left(k\right)\approx \int_{0}^{k}dk{\left(2k\right)}^{d-1}\pi \frac{\partial n}{\partial t}=\int_{0}^{k}dk{\left(2k\right)}^{d-1}\pi I\left(k\right) \end{array}$$ (32)

Dimensional analysis will give a similar relation as (Equation 31).


$$\begin{array}{l} Q\left(k\right)\approx k^{d}k^{2m-2s+d-\alpha }=k^{2m-2s+2d-\alpha } \end{array}$$ (33)

Assuming a constant flux of energy or wave action (i.e., independent of **k**) leads to steady-state solutions. The corresponding spectral exponents (or the decay exponentials) are denoted by *s*_*e*_ for energy and *s*_*n*_ for wave action, Equation 34.


$$\begin{array}{l} s_{n}=s_{e}-\frac{\alpha }{2} \end{array}$$ (34)

The distribution over the wave-frequency ω (Equation 35) can be estimated using Equations 31, 33 and the dispersion relation (Equation 10).


$$\begin{array}{l} n_{e}\left(\omega \right)=Ak^{-\alpha \frac{s_{e}}{\alpha }}=A\omega^{-\frac{m+d}{\alpha }}\\ n_{n}\left(\omega \right)=Ak^{-\alpha \frac{s_{n}}{\alpha }}=A\omega^{-\frac{m+d}{\alpha }+\frac{1}{2}} \end{array}$$ (35)

For the four-wave interaction, a similar set of relations can be derived, Equation 36. However, the collision integral *I* will have a different dimensionality compared to the three-wave interaction case.


$$\begin{array}{l} I\approx \int |V{|}^{2}n^{3}\delta \left(k\right)\delta \left(\omega \right)dk^{3}\\ \;\approx k^{2m+2d-3s-\alpha } \end{array}$$ (36)

The flux of the energy and wave action are given in Equation 37.


$$\begin{array}{l} P\left(k\right)\approx k^{d+\alpha }k^{2m+2d-3s-\alpha }\\ \;=k^{2m+3d-3s}\\ Q\left(k\right)\approx k^{d}k^{2m+2d-3s-\alpha }\\ \;=k^{2m+3d-3s-\alpha } \end{array}$$ (37)

The distribution over the wave frequency, ω, is given in Equation 38.


$$\begin{array}{l} n_{e}\left(\omega \right)=A\omega^{-\frac{\frac{2m}{3}+d}{\alpha }}\\ n_{n}\left(\omega \right)=A\omega^{-\frac{\frac{2m}{3}+d}{\alpha }+\frac{1}{3}} \end{array}$$ (38)

The expressions in Equations 35, 38 present the predicted spectral profiles for dynamics arising from 3- and 4-wave interactions. In Section 4.7, these theoretical predictions will be compared with experimentally observed electrical recordings from the cortex.

### 4.5 Single and dual cascade solutions of spectral decay

The steady states outlined above correspond to cascades of either energy or wave action. Such systems are commonly termed single-cascade systems, since the entire spectral distribution in the inertial range is controlled by the cascade of a single conserved quantity across different regions of *k*-space. Nevertheless, it is possible for different frequency bands to exhibit distinct power-law decays, a phenomenon that has been observed in empirical measurements of cortical activity (Miller et al., 2009). Systems that support multiple cascades will display several power-law regimes; for example, in dual-cascade systems, two conserved quantities undergo cascading at the same time. These have been analyzed and modeled by Kraichnan (1967).

Building on the results derived in Section 4.2, we can construct a dual-cascade model. Part of the cascade corresponds to an energy cascade and the other to a wave action cascade. We have observed that the difference in spectral gradient between these regimes is either $\frac{1}{2}$ or $\frac{1}{3}$, depending on whether the system is governed by three-wave or four-wave interactions.

From energy conservation principles, we expect an inverse wave action cascade from a given input scale *k*_0_ toward smaller wavenumbers, and a direct energy cascade toward larger wavenumbers, where the energy is dissipated. A characteristic increase in spectral gradient is observed in dual-cascade systems in fluid dynamics, where the inverse cascade is of energy and the direct cascade involves enstrophy (vorticity squared), transferring from small to large *k* (Yakhot and Zakharov, 1993; She and Jackson, 1993). In such systems, the energy input scale corresponds to the transition point in the spectral slope.

Theoretical estimates of the spectral gradients for these steady-state systems can be found in Boffetta (2007) and Kraichnan (1967). Unlike fluid dynamics, wave systems lack a direct analog to vorticity in their governing equations. However, it is possible to define a variable in wave dynamics that exhibits dynamical characteristics analogous to vorticity in fluid systems.

#### 4.5.1 Vortices in wave theory

Enstrophy arises from the conservation of vorticity in the dynamics. In fluid turbulence, the vorticity equation is obtained by taking the curl of the Navier-Stokes equation, which governs the dynamics of the fluid; see Equation 39.


$$\begin{array}{l} \frac{\partial }{\partial t}\text{u}+\text{u}\cdot \nabla \text{u}=-\frac{1}{\rho }\nabla p+\nu \nabla^{2}\text{u}\\ \;\omega =\nabla \times \text{u} \end{array}$$ (39)

In the 2D case we get a simplification of Equation 39.


$$\begin{array}{l} \frac{\partial }{\partial t}\omega +\omega \cdot \nabla \text{u}=\nu \nabla^{2}\omega \end{array}$$ (40)

The dynamical equations governing neural fields differ fundamentally from those in fluid dynamics and do not contain an intrinsic vorticity term (Equation 40). However, it can be shown that wave dynamics governed by complex fields—i.e., waves characterized by distinct amplitude and phase variables—can be transformed into a fluid-like dynamical framework. A commonly used complex dynamical equation in the modeling of neural fields is the nonlinear Schrödinger equation, which captures key features of wave propagation and interaction in such systems, Equation 41.


$$\begin{array}{l} i\partial_{t}\phi =-\nabla^{2}\phi +|\phi {|}^{2}\phi \end{array}$$ (41)

This equation can be transformed into a fluid-like dynamical form using the Madelung transformation, as demonstrated in Spiegel (1980). By expressing the fields of the nonlinear Schrödinger equation in terms of amplitude and phase variables, and performing the appropriate differentiations, one obtains a dynamical equation closely resembling the Navier-Stokes equation, Equations 42, 43, 44.


$$\begin{array}{l} \phi =\sqrt{\rho }e^{i\psi } \end{array}$$ (42)

In this formulation, the fluid density is defined as ρ, and the velocity field is given by **u** = 2∇ψ, where ψ denotes the phase of the complex wave function. Importantly, the velocity arises from the gradient of the phase variable, highlighting the correspondence between wave phase dynamics and fluid motion. The above transoformation transforms the euqation describing the activity of the cortical surface into a model with phase oscillators with a source term (which estimates the amplitude of the activity), similar to the Kuramoto model (Breakspear et al., 2010; Kuramoto, 1997). The presence of sources of phase rotations on the cortex will then generate vortices in the above model. In the supplementary material, a simulation is presented based on the model introduced in this paper, with the velocity field plotted to demonstrate the emergence of vortices. This behavior requires that the coupling between neuronal units include a rotational component, analogous to the mechanism described in Cooray et al. (2025). Similar vortical patterns have also been reported in multiple experimental datasets, using both invasive and non-invasive recordings of human brain activity (Xu et al., 2025; Bhattacharya et al., 2022; Dickey et al., 2021; Muller et al., 2016).


$$\begin{array}{l} \frac{Du}{Dt}=\partial_{t}\text{u}+\left(\text{u}\cdot \nabla \right)\text{u}=-\frac{\nabla \rho^{2}}{\rho }+2\nabla \frac{\nabla^{2}\sqrt{\rho }}{\sqrt{\rho }}\\ \;\frac{\partial \rho }{\partial t}+\nabla \cdot \left(\rho \text{u}\right)=0 \end{array}$$ (43)

The resulting equation corresponds to a variation of Euler flow for compressible fluids–specifically, an isentropic compressible Euler flow with an adiabatic index of 2 (Nazarenko, 2011). The final term on the right-hand side is often referred to as the *quantum pressure* term, due to its origin in the Schrödinger equation. Taking the curl of this equation yields the vorticity equation, thereby linking the phase structure of wave dynamics to rotational features in fluid-like systems.


$$\begin{array}{l} \frac{D\omega }{Dt}=\left(\omega \cdot \nabla \right)\text{u}-\omega \left(\nabla \text{u}\right)+\frac{\nabla \rho \times \nabla \rho^{2}}{\rho^{2}} \end{array}$$ (44)

Similarly, the Klein-Gordon field, including the connectivity gauge, can be transformed into a hydrodynamical form exhibiting vorticity (Takabayasi, 1953; Cooray et al., 2025). The spectral index for the vorticity cascade can be estimated using a relation from Kraichnan (1967). In particular, the vorticity cascade index *a*_ω_ (that is, the power-law decay exponent) is connected to the energy cascade index *a*_*e*_ through the following relation, Equation 45:


$$\begin{array}{l} a_{\omega }=\frac{1}{2}\left(3a_{e}+1\right) \end{array}$$ (45)

### 4.6 Multifractal state

The steady-state solutions outlined above rely on the Richardson cascade (Kolmogorov, 1991; Frisch and Kolmogorov, 1995), in which energy is passed from larger eddies to smaller ones, with each eddy occupying an equal spatial volume. The spatial dimensionality of these eddies is usually 2 or 3, depending on whether the fluid is constrained to a plane or moves within a three-dimensional volume. Empirical measurements of the power-law decay on the cortex, however, are not consistent across studies (He, 2014; Miller et al., 2009; Sheremet et al., 2019). As discussed in more detail below, the concept of a multifractal state nevertheless permits variability in the spectral power-law decay.

In multifractal models of turbulence (Frisch and Kolmogorov, 1995), the energy-containing structures do not fill the entire spatial domain but are instead supported on fractal sets characterized by a non-integer Hausdorff dimension. The Hausdorff dimension is a measure of the fractal geometry of a set, quantifying how its detail changes with scale and capturing its complex, self-similar structure.

A similar fractal dynamics can be assumed for wave turbulence, where the family of interacting eddies or waves occupies a fractal subset of the *k*-space. This fractal support alters the spectral indices of the Kolmogorov-type solutions, as demonstrated in Equation 46.


$$\begin{array}{l} a_{e}=\frac{m+2d-D}{\alpha }\\ a_{n}=\frac{m+2d-D}{\alpha }-\frac{1}{2} \end{array}$$ (46)

The dimension of the fractal set to which the dynamics of the interactions are attracted is denoted by *D*. When this dimension equals the full spatial dimension *d*, the cascade dynamics reproduce the original Kolmogorov spectra. As *D* decreases toward zero, the spectral slope becomes progressively steeper.

It is also possible for the dynamics to be attracted to different fractal structures simultaneously. In such a *multifractal* state, characterized by a spectrum of fractal dimensions, the Kolmogorov spectra are modified so that the constant power-law decay may either increase or decrease as *k* tends to zero or infinity (Frisch and Kolmogorov, 1995; Mandelbrot, 1974; Benzi et al., 1984; Sreenivasan, 1997).

### 4.7 Wave turbulence and steady state spectra

Experimental recordings of cortical activity have been performed in various animal models as well as in humans. In humans, such recordings are routinely used in the clinical assessment of cortical function, primarily for the diagnosis and monitoring of epilepsy. Recordings are obtained either non-invasively, using sensors placed on the scalp (electroencephalography, EEG), or invasively, with electrodes placed intracranially—either on the cortical surface (subdural electrodes) or within the cortical tissue itself (stereo-EEG).

All of these recordings exhibit a characteristic frequency spectrum featuring an inverse power-law decay of the form $\frac{1}{f^{a}}$ over approximately the 1-100 Hz range, often referred to as the inertial region in wave turbulence terminology. This power-law component is also known as the aperiodic component of the EEG (Donoghue et al., 2022). The exponent *a* has been estimated in humans to lie roughly between 2 and 3. This steady-state spectral feature is observed across different brain states, including both wakefulness and sleep.

**Figure 2:**
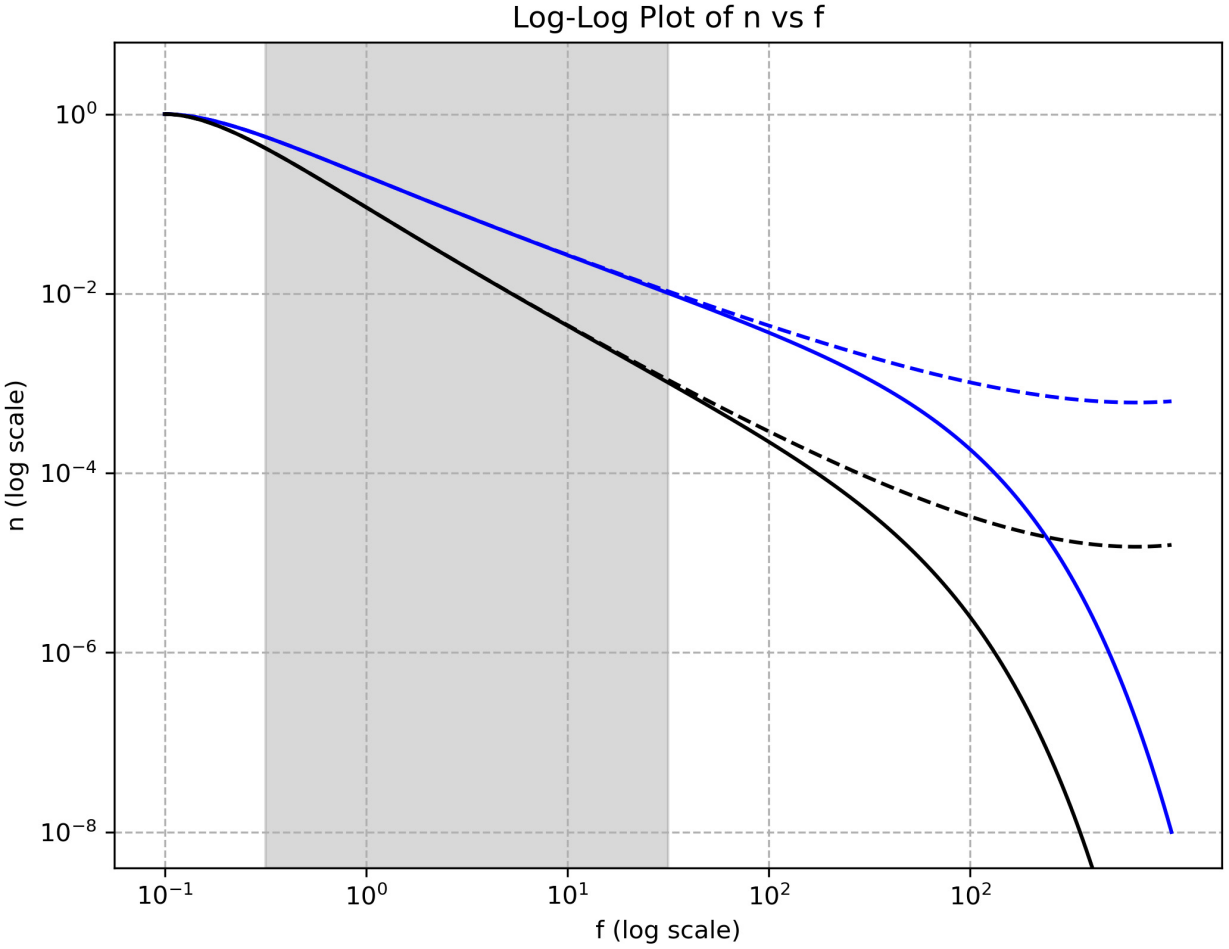
Log-log plot of the squared amplitude versus frequency observed in cortical activity. Within the shaded gray region, the spectral features exhibit a linear decay with a slope varying between 2 and 3. This gray region, corresponding to the inertial range of the wave dynamics, spans approximately from 1 to 100 Hz. At higher frequencies, the spectral data either decay rapidly (solid lines) or saturate to a constant level (dashed lines). At lower frequencies (to the left of the gray region), the spectral features also exhibit saturation.

Experimental data also reveal changes in spectral features at higher frequencies (above 100–200 Hz), where the spectrum either decays rapidly or saturates at a minimal level. Figure 2 illustrates a schematic overview of the different spectral patterns observed in cortical tissue recordings.

**Figure 3:**
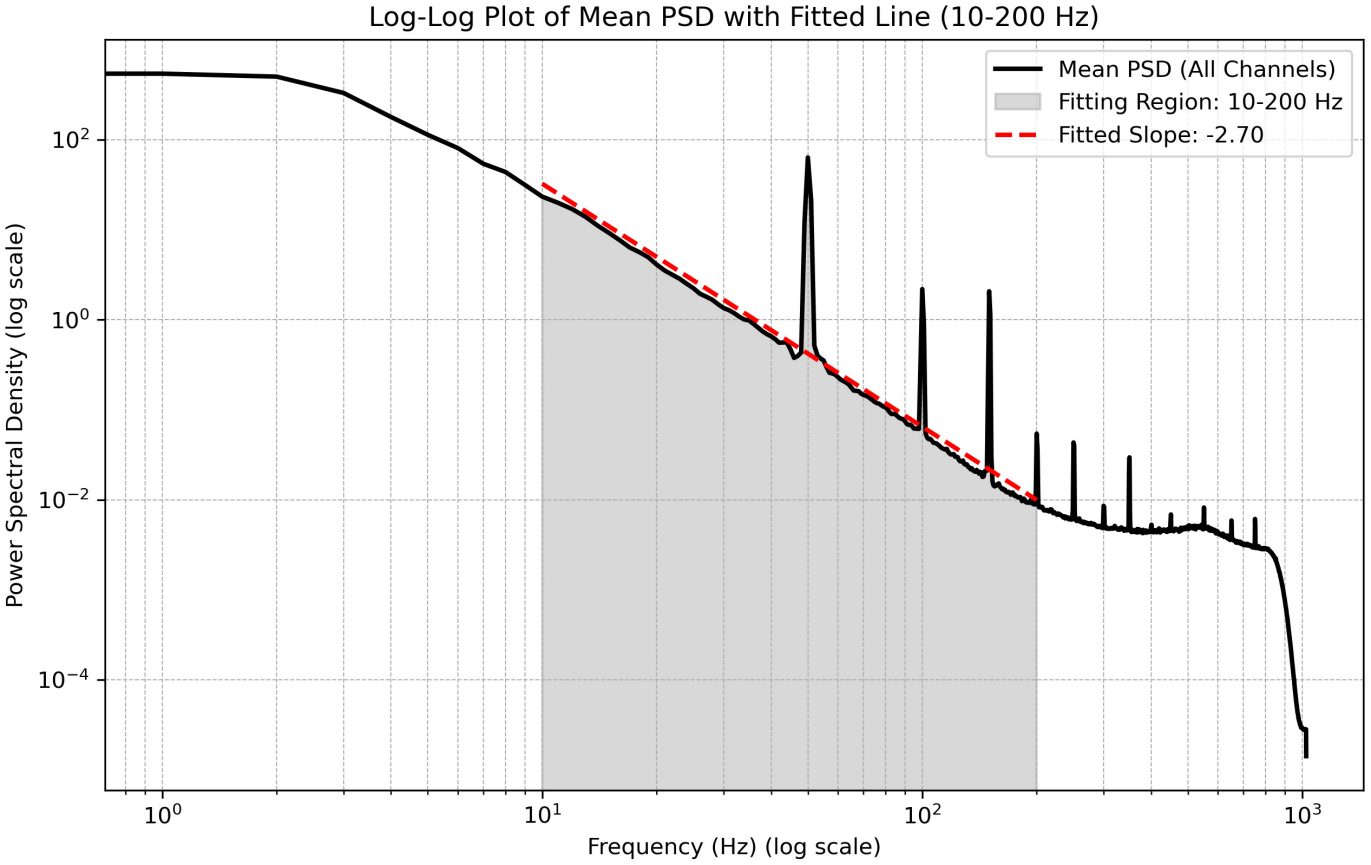
Spectral features of cortical activity recorded using intracranial electrodes. The central region (shaded in gray) exhibits a power-law decay with an estimated slope of approximately −2.7. Outside this region, the spectrum saturates, followed by a sharp decay near 1,000 Hz. Note the presence of artifactual spikes at 50 Hz and its harmonics.

Figure 3 shows empirical estimates of the spectral features, highlighting a central inertial region with a characteristic power-law decay. Outside this inertial range, the spectral features saturate as frequencies increase or decrease. Assuming a frequency power-law decay with an exponent between 2 and 3, we can estimate certain features of the underlying model. Considering the classical Kolmogorov spectra (i.e., without fractal subdimensions), the following relations hold for three-wave interactions, Equation 47:


$$\begin{array}{l} a_{e}=\frac{m+d}{\alpha }\approx \frac{m+2}{1} \end{array}$$ (47)

We have assumed an approximately linear relation between the wave frequency and momentum (α ≈ 1). The scaling parameter *m* is difficult to define precisely due to uncertainty about which model best describes the overall dynamical behavior of neural fields. However, it can be argued that *m* may be estimated using the above relations. In fluid dynamic turbulence, where the underlying dynamical equations are better understood, a value of *m* = 0.5 is commonly used. This corresponds to a power-law decay exponent of approximately −2.5, consistent with the empirical observations shown in Figure 3. For four-wave interactions, a slightly smaller exponent is predicted according to Equation 48.


$$\begin{array}{l} a_{e}=-\frac{\frac{2m}{3}+d}{\alpha }\approx -\frac{\frac{2m}{3}+2}{1} \end{array}$$ (48)

The above equations involve two unknown variables: the scaling factor *m* and the exponent α. The multifractal dynamics described in Section 4.4 yield a similar expression but include an additional variable representing the fractal dimension of the structure containing the eddies. In general, the resulting steady-state spectra are steeper.

The multifractal model also accounts for the saturation of the wave action *n* at low frequencies (or small *k* values), as well as the rapid decay or saturation observed at high *k* values.

All scenarios discussed so far in this section involve a single cascade within the inertial range. However, several studies have reported spectral features consistent with a dual cascade. Specifically, two distinct regions with different power-law decay slopes have been observed (Miller et al., 2009). The decay exponent at high frequencies was approximately −4, whereas for smaller *k* values it was around −2.5.

**Figure 4:**
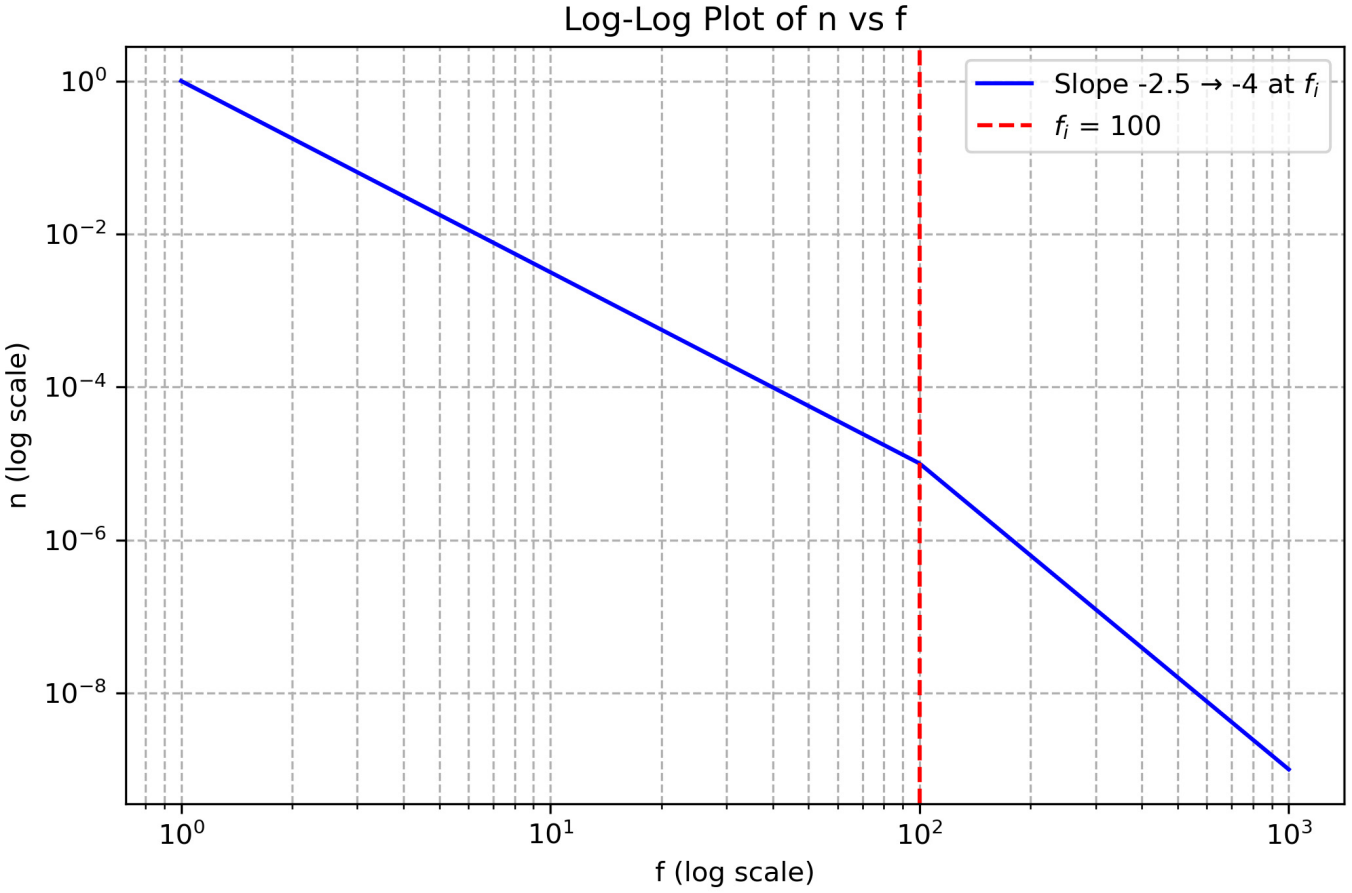
Double cascade system with an inverse energy cascade below 100 Hz with a decay exponent of approximately −2.5. Direct cascade is seen above 100 Hz involving vorticity transfer to higher frequencies. Empirical data support these characteristic decay values.

Equation 45 predicts a decay exponent of −4 for the second cascade if the first energy cascade has a decay exponent of −2.5, consistent with the findings illustrated in Figure 4.

## 5 Sources and sinks of the steady state

The Kolmogorov solution, which assumes a constant energy flux, requires the presence of both a source and a sink to sustain a non-zero flux. This, in turn, means that the underlying model must be non-conservative, a condition that is typically taken to hold for neural fields. The central assumption for deriving steady-state solutions is that the rate of energy attenuation by the dynamics depends on the frequency content of the activity. When this is approximately true, the resulting steady-state solutions accurately capture the behavior of the system between the source and sink. In *k*-space, the source and sink regions are generally well separated. The region of k-space in which dissipative forces exceed the non-linear conservative forces—that is, the sinks—will typically occur at the upper end of the k-spectrum. As outlined above, the dissipative forces are not modeled explicitly, but only implicitly via the sink term Γ, as defined in equation 49. Based on the spectral characteristics of the recorded data, the upper part of the inertial range (i.e. the portion characterized by a power-law decay) corresponds to a regime where the dissipative forces become comparable to the conservative forces. In Figure 3, the decay of the spectral amplitude gradually shifts from a power-law form to a dissipative decay at around 100 Hz. At this frequency, assuming a wave speed of approximately 10 *mms*^−1^, the associated length scale is about 0.1 *mm*. This is on the order of the size of cortical columns generating the activity, implying that the microscales of the model—specifically, the scale at which dissipative forces dominate over conservative forces—lie within the range of the constituent building blocks of cortical architecture. This is analogous to the Taylor and Kolmogorov microscales in hydrodynamics.

In this section, we investigate how the presence of sources and sinks modifies the Kolmogorov steady state. The spectral characteristics of EEG recordings exhibit several features that can inform the nature of the underlying dynamics. We will examine the effects of both wideband and narrowband sources, as well as the impact of high-frequency damping on steady-state solutions. The source/sink term, denoted by Γ, modifies the governing expressions developed in Sections 3 and 4, Equation 49.


$$\begin{array}{l} \frac{\partial n\left(k,t\right)}{\partial t}=I\left(n\left(k\right)\right)+n\left(k\right)\Gamma \left(k\right) \end{array}$$ (49)

Note that the Kolmogorov spectrum retains the same power-law decay rate within the inertial range, where the source/sink term Γ is assumed to be zero. In this regime, the energy flux is conserved and the spectral shape remains unaffected by the presence of sources or sinks located outside the inertial range.

### 5.1 Effect of narrowband sources on the spectrum

The impact of a broadband source was shown in Zakharov et al. (1992) to preserve the spectral power-law decay, altering the spectrum only by an overall multiplicative constant. In contrast, a narrowband source can substantially modify the spectrum. A narrowband source can be mathematically represented as a Dirac delta function δ(*k*_0_), centered on the injection scale *k*_0_, Equation 50.


$$\begin{array}{l} \Gamma \left(k,t\right)=\Gamma_{0}\delta \left(k_{0}\right) \end{array}$$ (50)

The effect of a narrowband source on the spectrum can be estimated qualitatively and will depend on the specific form of nonlinearity in the underlying Hamiltonian of the system. For a three-wave interaction nonlinearity, the result is a cascade of spectral spikes superimposed on the underlying Kolmogorov spectrum. Let *n*_1_(*k*) denote the original Kolmogorov spectrum and *n*_2_(*k*) the source-induced modulation, Equation 51. The total spectrum is then given by the combination of these two components, where the presence of *n*_2_(*k*) introduces localized spikes at harmonics of the source frequency.


$$\begin{array}{l} n\left(k,t\right)=n_{1}\left(k,t\right)+n_{2}\left(k,t\right) \end{array}$$ (51)

Nonlinear interactions between source-induced spikes generate recurrent spectral features at integer multiples of the injection scale *k*_0_, i.e., at *k* = *nk*_0_ for integer *n*, Equation 52.


$$\begin{array}{l} n_{2}\left(k,t\right)=\underset{j}{\sum }a_{j}\delta \left(jk_{0}\right) \end{array}$$ (52)

As a first approximation, it can be assumed that the activity (or amplitude) within each spike follows the same decay as the Kolmogorov spectrum, Equation 53.


$$\begin{array}{l} n_{2}\left(k,t\right)=\underset{j}{\sum }j^{-\left(m+d\right)}k_{0}^{-\left(m+d\right)}\delta \left(jk_{0}\right) \end{array}$$ (53)

However, each spectral peak will exhibit a finite width, denoted by Δ*k*_0_, which increases with each successive cascade of the spikes. This broadening can be understood intuitively, as the system involves a two-wave to one-wave interaction process, leading to increasing spectral dispersion at higher harmonics, Equation 54.


$$\begin{array}{l} k_{0}\pm \delta k\to 2k_{0}\pm 2\delta k \end{array}$$ (54)

Each spike can be estimated as a triangular region, Equation 55.


$$\begin{array}{l} \delta \left(k_{0}\right)\to \Delta \left(k_{0},\delta k\right) \end{array}$$ (55)

The interactions will lead to a cascading set of spikes, Equation 56.


$$\begin{array}{l} n_{2}\left(k,t\right)=\underset{j}{\sum }j^{-\left(m+d\right)-1}k_{0}^{-\left(m+d\right)}\Delta \left(jk_{0},j\delta k\right) \end{array}$$ (56)

The full spectra will be given in Equation 57.


$$\begin{array}{l} n\left(k,t\right)=\lambda P^{\frac{1}{2}}k^{-\left(m+d\right)}+\underset{j}{\sum }j^{-\left(m+d\right)-1}k_{0}^{-\left(m+d\right)}\Delta \left(jk_{0},j\delta k\right) \end{array}$$ (57)

**Figure 5:**
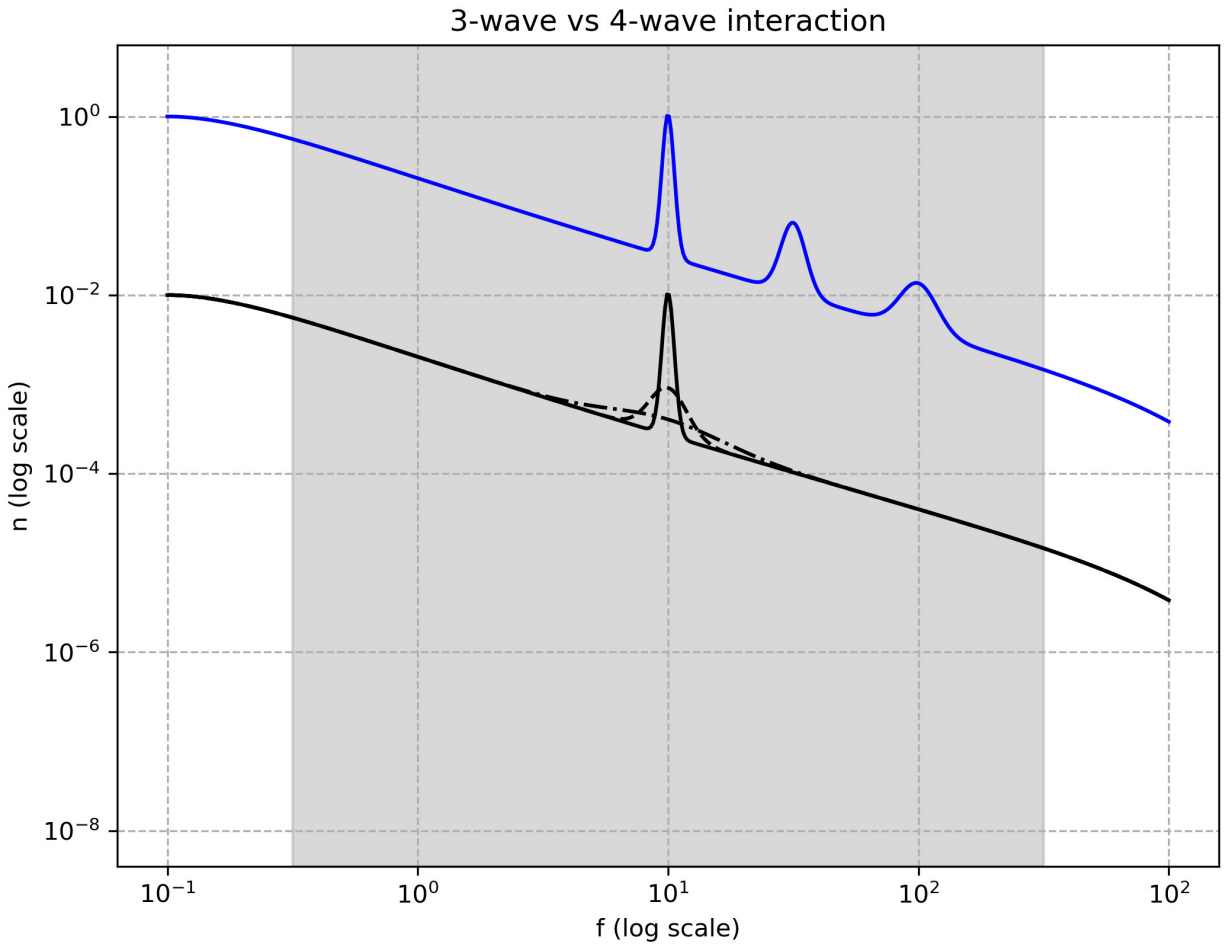
Narrow spectral pumping in three-wave (blue) and four-wave (black) turbulence interactions. In the three-wave interaction regime, harmonic peaks emerge at integer multiples of the pumping frequency. These harmonics are generated through a process in which two waves within the pumped region combine to form a new wave with approximately twice the frequency. This interaction recurs iteratively, with higher-order harmonics arising from successive interactions between previously generated peaks. In contrast, four-wave interactions exhibit a fundamentally different behavior: two waves within the pumped region scatter off each other, generating wave pairs with a range of frequency ratios. This results in a progressive temporal dispersion of the pumped spectral activity rather than the formation of discrete harmonics.

Experimental and numerical simulations have provided evidence supporting a cascade of spectral spikes consistent with three-wave interaction dynamics (see Figure 5; Sheremet et al., 2019; Donoghue et al., 2022). In contrast, four-wave interactions result primarily in the dispersion and broadening of spectral peaks over time, a phenomenon that has also been observed in some datasets (Hirai et al., 1999).

### 5.2 Transient cortical activity

In the preceding sections, we examined the effects of continuous sources on cortical activity. In this section, we investigate the impact of short-lived, transient sources on the dynamics of cortical activity. The partial differential equation that governs the system under these conditions is given by Equation 58.


$$\begin{array}{l} \frac{\partial n\left(k\right)}{\partial t}=I\left(n\left(k\right)\right)+n\left(k\right)\Gamma \left(k,t\right) \end{array}$$ (58)

A Dirac spike will be used to model a short initial source, Equation 59.


$$\begin{array}{l} \Gamma \left(k,t\right)=\delta \left(t-t_{0}\right)\delta \left(k-k_{0}\right) \end{array}$$ (59)

This yields the following expression (Equation 60), neglecting the first term on the right-hand side.


$$\begin{array}{l} \;\frac{\partial n_{k}}{\partial t}=n_{k}\delta \left(t-t_{0}\right)\delta \left(k-k_{0}\right)\\ \frac{1}{n_{k}}\frac{\partial n_{k}}{\partial t}=\delta \left(t-t_{0}\right)\delta \left(k-k_{0}\right)\\ \ln\;n_{k}=\Theta \left(t-t_{0}\right)\delta \left(k-k_{0}\right) \end{array}$$ (60)

The resulting waveform will be concentrated around the site of energy insertion, Equation 61.


$$\begin{array}{l} n\left(k_{0},t\right)>>n\left(k,t\right) \end{array}$$ (61)

There is a significant elevation in wave action, *n*, at the site of energy introduction compared to other regions in *k*-space. However, the collision integral encompasses dynamical processes that allow for the redistribution of this introduced activity across *k*-space. In the previous section, we described how 3-wave interactions can generate higher-order spectral spikes at integer multiples of *k*_0_. One aspect not previously discussed is the role of disintegrative interactions, where a single wave splits into two daughter waves with reduced momentum and energy. As there is no strict constraint on the energy ratio between these resulting waves, this mechanism allows for a diffusion of spectral energy toward lower *k* values—particularly on the lower-frequency (left) side of the induced spike. The corresponding collision integral governing this process is provided in Equation 16. To investigate the dynamics on the lower-*k* (left) side of the spectral spike, we consider the scattering processes in which the waves are redistributed into slightly smaller *k* values. This corresponds to interactions that result in the transfer of energy or wave action from the vicinity of the spike toward lower frequencies, Equation 62.


$$\begin{array}{l} \;1\to k+2\\ n\left(k_{1}\right)=n\left(k\right)+\delta n\\ n\left(k_{2}\right)=\delta n \end{array}$$ (62)

Expanding the collision integral given in Equation 16, we obtain (Equation 63).


$$\begin{array}{l} I\left(n\left(\text{k}\right)\right)=\frac{\partial n\left(\text{k}\right)}{\partial t}=\\ \;\pi \int [|K_{1}\left(\text{k},{\text{k}}_{1},{\text{k}}_{1}\right){|}^{2}N_{1}\left(\text{k},{\text{k}}_{1},{\text{k}}_{2}\right)\\ \;+2|K_{1}\left({\text{k}}_{1},\text{k},{\text{k}}_{1}\right){|}^{2}N_{1}\left({\text{k}}_{1},\text{k},{\text{k}}_{2}\right)]d{\text{k}}_{1}d{\text{k}}_{2}\\ \;\approx 2|K\left({\text{k}}_{1},{\text{k}}_{1},{\text{k}}_{2}\right){|}^{2}n{\left(k\right)}^{2} \end{array}$$ (63)

With further simplification, we can estimate how the amplitude evolves on the lower-*k* side of the spike, Equation 64.


$$\begin{array}{l} \;\frac{\partial n\left(k\right)}{\partial t}=Cn^{2}\left(k\right)\\ \;\frac{dn\left(k\right)}{n^{2}\left(k\right)}=Cdt\\ A-\frac{1}{Ct}=n\left(k\right) \end{array}$$ (64)

We notice an enhancement of wave activity to the left of the spike, suggesting that this portion of the spectral deformation causes the spike to be deflected toward lower *k*-values. To evaluate how small perturbations develop in this region, one can employ the linearised version of the collision integral (Zakharov et al., 1992). It can then be shown that the perturbations evolve as described in Equation 65.


$$\begin{array}{l} \frac{\partial \delta n_{k}}{\partial t}\approx \\ \;-|K\left(k_{1},k,k_{2}\right){|}^{2}n\left(k_{2}\right)\frac{\partial \delta n\left(k\right)}{\partial k} \end{array}$$ (65)

This equation represents a wave propagating to the left with velocity $c=|K\left(k_{1},k,k_{2}\right){|}^{2}n\left(k_{2}\right)$. We take *n*(*k*_2_) to be constant, since it is small and located in a part of the spectrum well separated from the perturbative bump on the left. Employing the dispersion relation, the corresponding frequency decrease can be estimated in the same manner as in Equation 64, resulting in a reduction in frequency as described below, Equation 66.


$$\begin{array}{l} \omega \left(k\right)=A{\left(B-|K\left(k_{1},k,k_{2}\right){|}^{2}n\left(k_{2}\right)t\right)}^{\alpha }\\ \frac{\partial \omega \left(k\right)}{\partial t}=-\alpha A|K\left(k_{1},k,k_{2}\right){|}^{2}n\left(k_{2}\right){\left(B-|K\left(k_{1},k,k_{2}\right){|}^{2}n\left(k_{2}\right)t\right)}^{\alpha -1} \end{array}$$ (66)

**Figure 6:**
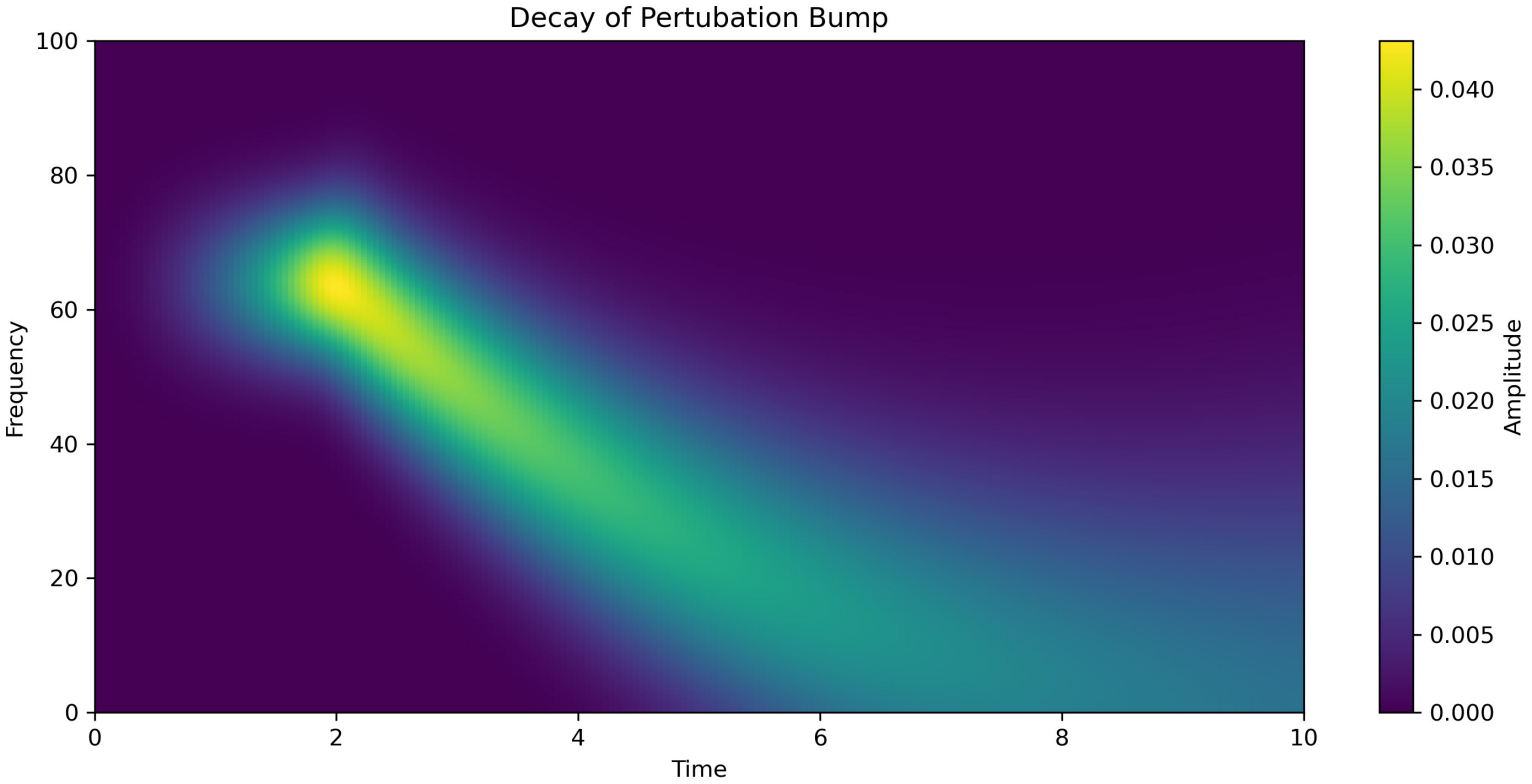
Estimation of the decay of a perturbative bump around the steady-state solution reveals a power-law decay in frequency, accompanied by dispersion of the signal. The time-frequency plot of EEG activity changes during seizure onset exhibits a similar pattern, often referred to as high-frequency, low-amplitude seizure onset activity.

A comparison with empirically observed frequency variations during electrographic seizures recorded at the cortical surface shows a comparable temporal profile. The simulated time course of seizure frequency mirrors this pattern, as illustrated in Figure 6 (Lagarde et al., 2019).

## 6 Discussion

In this paper, we discussed the theoretical foundation of wave turbulence and its relevance to the analysis of neural fields. We derived key statistical features of the underlying dynamics, including the characteristic power-law decay that is consistently observed in recordings from cortical tissue. A range of possible spectral patterns was presented, along with the theoretical mechanisms that may generate them. These theoretical predictions were compared with representative examples from the literature, highlighting matching spectral features observed in empirical data.

The model presented is a combination of a non-linear conservative model together with non-conservative dyanmics added to the conservative model. Which is of importance as this allows for the creation of steady state soloutins giving spectral feuatres similar to what is seen empirically. The source of the non-conservative dynamics is present both at the intrinsic dynamics of the neuronal systems (described in section 2) but also as the presence of long term connections which would transfer and move energy of the local system to and from other parts of the brain. Furthermore, subcortical afferant activity to the cortex e.g. thalamic input in the human brain, would be modeled as a pumping source.

A comprehensive analytical treatment of turbulence was first developed by Kolmogorov in 1941, building on the experimental observations available at the time. For fluid turbulence, a power-law decay of energy with respect to wave-number was predicted, characterized by an exponent of $\frac{5}{3}$. This result has been extensively verified in various experimental settings; for a comprehensive introduction and further references, see Frisch and Kolmogorov (1995).

Interestingly, similar power-law decay is a robust feature of recordings from cortical tissue, where the observed spectral exponent is typically higher, estimated between 2 and 3. While a direct link between classical fluid dynamics and the dynamics of cortical tissue is not immediately evident, there has long been a suggestion that the brain supports wave-like interactions. This idea is foundational to the theory of wave turbulence, notably described by Zakharov et al. (1992). The precise spectral decay rate in wave turbulence depends on the specific interaction model, and it has been proposed that models of cortical dynamics might be constrained or identified by matching their predicted decay rates to those empirically observed.

However, the range of theoretical models capable of producing spectral decays within the empirically observed range is quite broad. Furthermore, the spectral patterns observed in neural recordings are more intricate than the classical turbulence treated by Kolmogorov. The presence of chaotic or fractal attractors in the dynamics introduces additional parameters—such as the Hausdorff dimension—that further influence the decay rate. In fact, most nonlinear models can be tuned to produce either classical Kolmogorov spectra or multifractal decays (Benzi et al., 1984; Meneveau and Sreenivasan, 1991).

The literature on recordings from cortical tissue is notably variable. Several differences can be attributed to recording methodologies, as the size and configuration of the recording electrode significantly influence the data. However, when local field potentials (LFPs) or macroelectrodes sample activity from a sufficiently large neural population, the dynamics tend to be oscillatory rather than spiking. Spectral analysis of such oscillatory signals often reveals a power-law decay of amplitude with frequency. Some studies report a single power-law regime, while others identify two distinct regimes—a phenomenon consistent with either a single energy cascade or a dual cascade in turbulent systems (Deco and Kringelbach, 2020; Miller et al., 2009).

Moreover, cortical spectral activity frequently exhibits a combination of aperiodic (power-law) and periodic features. From the perspective of wave turbulence, periodic features could be interpreted as spectral pumping. The consequences of such pumping differ across studies: in some cases, the pumped activity disperses; in others, it generates harmonics. This discrepancy may reflect differences in the underlying wave interaction mechanisms. Specifically, systems governed by 3-wave interactions are known to produce harmonics, whereas 4-wave interactions tend to cause dispersion of energy introduced via pumping.

Determining the underlying interaction model is challenging, as empirical features point to multiple theoretical frameworks. For example, the nonlinear Schrödinger equation (NLSE) represents a canonical 4-wave interaction system. However, it is known that systems governed by the NLSE can undergo dynamical transitions–akin to Bose-Einstein condensation–into effective 3-wave systems (Pitaevskii, 1961). Currently, the available spectral data are insufficient to conclusively identify the governing dynamics of cortical fields.

Given that there is still no complete analytical understanding of turbulence in fluid or wave systems–despite decades of study and well-defined first-principles models–it is overly optimistic to expect a full derivation of neural field dynamics solely from spectral data. Nevertheless, certain features must be incorporated into any plausible model. These include the capacity for both 3-wave and 4-wave interactions, and the potential for either single or dual cascade phenomena, as observed in empirical data.

This paper explored cortical dynamics through the framework of wave turbulence. By drawing analogies with fluid turbulence, we examined how spectral features such as power-law decay, energy cascades, and non-linear interactions may arise from wave-based neural activity. While a complete analytical model of neural turbulence remains out of reach, the observed features constrain the space of viable models. Future work should aim to further link theoretical dynamics with measurable spectral characteristics, potentially revealing deeper insights into the principles governing large-scale brain activity.

## Data Availability

The original contributions presented in the study are included in the article/Supplementary material, further inquiries can be directed to the corresponding author.
