# Supplementary material for: Wave turbulence and cortical dynamics
Source: Front Comput Neurosci. 2026 Mar 20;20:1682176. doi: 10.3389/fncom.2026.1682176 (PMC13047063; doi:10.3389/fncom.2026.1682176)
Supplement: Supplementary file 1 [file Data_Sheet_1.pdf]

Supplemental data

Nomenclature

**Field Parameters**	
$\boldsymbol{r}, \boldsymbol{r'}, \boldsymbol{r_0}, \boldsymbol{r_1}$	Points on the cortical surface
δ	Variation
$\delta(x), \delta(k)$	Dirac delta function
$\lvert\mathbf{k}\rvert$	Norm or absolute value
$\mathbf{A}$	Connectivity field
$\mathbf{k}$	Momentum vector, conjugate to $\mathbf{r}$
$\mathcal{F}$	Complex Functional of the Neural Field
$\mathcal{H}_{int}$, $\mathcal{H}_0$,$\mathcal{H}_3$,$\mathcal{H}_4$	Hamiltonian functions
$\mathcal{M}$	Cortical manifold
ω	Angular velocity
$\phi(\boldsymbol{r}, t)$	Complex Neural Field at r and t
ϕ^*, ψ^*	Complex conjugate of the Field
Θ	Heaviside function
g	Non-linear coupling strength
i	Imaginary unit, $i^2 = -1$
i, j, k	Indexes depending on context.
K_0	First order Kernel Function of $\mathcal{F}$
K_1	Second order Kernel Function of $\mathcal{F}$
K_2	Third order Kernel Function of $\mathcal{F}$
t, t', t_0, t_1	Time points
**Differentials and Integrals**	
$\frac{\delta}{\delta\psi}$	Functional derivative
$\frac{D}{Dt}$	Directional derivative
$\int dt, \iint dr_1 dr_2$	Integral with corresponding integral measure
$\langle\psi\rangle$	Averaging of the field variable ψ
∇	Partial differential in space
∂_t	Partial time derivative
$\tilde{\phi}$	Adiabatic time average of a function.
**Fourier Transforms**	
$\mathcal{K}_0$	Fourier transform of the first order kernel function of $\mathcal{F}$
$\mathcal{K}_1$	Fourier transform of the second order kernel function of $\mathcal{F}$
$\mathcal{K}_2$	Fourier transform of the third order kernel function of $\mathcal{F}$
$\psi(\boldsymbol{k}, t)$	Spatial Fourier transform of ϕ or $\tilde{\phi}$

Turbulence Parameters

α	Dispersion coefficient
$\boldsymbol{\omega}$	The vorticity field
Γ	Energy source/sink term
λ	Scaling constant
$\mathbf{e}_\theta$	A unit vector in the azimuthal direction
$\mathbf{u}$	A velocity field derived after the Madelung transformation
μ	Constant
ρ	Density field
A, B, C	Constants
d	Dimension of the space where cortical turbulence occurs
E, N	Total energy and wave action of oscillatory waves
I	Collision integral
m	Power of scaling law
$n(\mathbf{k})$	Amplitude for waves with momentum, $\mathbf{k}$
$P(k)$	Energy flux at wave momentum, k
$Q(k)$	Wave action flux at wave momentum, k
S	Entropy
s	Scaling law coefficient

1 Vortex Solution and Phase-Driven Dynamics

Stationary solutions of the four-wave Hamiltonian $\mathcal{H}_4$, augmented by a connectivity field, were simulated. The corresponding equation of motion is given in Eq. (1). The connectivity field encodes the couplings between neural units across the cortical field.

$$i\frac{\partial \psi}{\partial t} = -\frac{1}{2}(\nabla - i\mathbf{A})^2\psi + g|\psi|^2\psi \tag{1}$$

The biophysical source of this rotational vortex is the connectivity field $\mathbf{A}$, as defined in [1]. In [1], cortical dynamics are described as arising from the interaction between the activity of neuronal units and the connectivity that links them. Analogous to neuronal activity, the connection gain between neuronal units is characterized by both an amplitude and a phase. For our simulations, we specified the connectivity field $\mathbf{A}$ in Eq. (2). Note that A corresponds to the angular speed of a rotating system with equivalent dynamics.

$$\mathbf{A} = A\begin{pmatrix} -y \\ x \end{pmatrix} \tag{2}$$

The computational domain was discretised on a uniform square grid, and the Laplacian was approximated using second-order finite differences. An initial condition with a vortex was defined as in Eq. (3).

$$\psi = \tanh(r)e^{i(\theta+\alpha r)} \tag{3}$$

The velocity and vorticity field of Eq. (3) is given by the following conditions (using the Madelung transformation, see section 4.5.1 in the main manuscript):

$$\begin{aligned}\mathbf{u} &= \frac{1}{r}\mathbf{e}_\theta \\ \boldsymbol{\omega} &= \nabla \times \mathbf{u} \\ &= 2\pi\delta(\mathbf{r})\end{aligned} \tag{4}$$

This state was propagated forward in time using a split-step Fourier method. The resulting dynamics revealed how the amplitude $|\psi|^2$ interacts with the phase gradient of the state. The central dip in activity travels outward along the phase flow, as illustrated in figure 1. More complex behavior can be modeled by introducing multiple interacting vortices governed by the cortical dynamics.

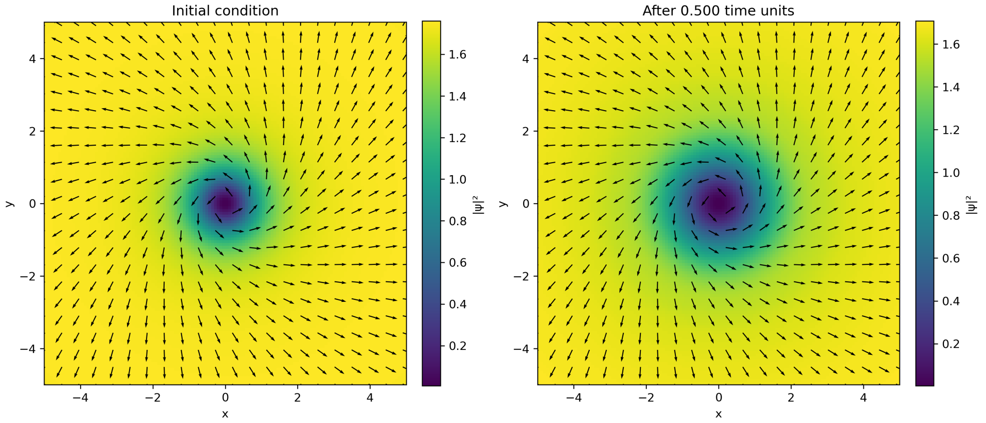

Fig. 1 The panel on the left displays the initial condition, which was simulated using the 4-wave interaction model. As the dynamics evolve, the right panel illustrates how the reduced amplitude at the origin propagates outward.

References

[1] Cooray, G.K., Cooray, V., Friston, K.J.: Cortical dynamics of neural-connectivity fields. Journal of Computational Neuroscience, 1–19 (2025)
